# *Giardia duodenalis* in Hu sheep: occurrence and environmental contamination on large-scale housing farms[Fn FN1]

**DOI:** 10.1051/parasite/2023004

**Published:** 2023-01-25

**Authors:** Qianming Zhao, Chenyang Lu, Zhiyang Pei, Pihong Gong, Junqiang Li, Fuchun Jian, Bo Jing, Meng Qi, Changshen Ning

**Affiliations:** 1 College of Animal Science and Technology, Tarim University Alar 843300 Xinjiang PR China; 2 College of Veterinary Medicine, Henan Agricultural University Zhengzhou 450046 Henan PR China

**Keywords:** *Giardia duodenalis*, Infection rates, Multilocus genotypes, Large-scale sheep farm, Feces, Environmental

## Abstract

*Giardia duodenalis* is a common zoonotic intestinal parasitic protozoan and sheep are among its hosts; however, limited information is available on sheep kept in large-scale housing. The Hu sheep is a first-class protected local livestock breed in China. In this study, we investigated the seasonal dynamics of *G. duodenalis* infection in Hu sheep and the environmental contamination of large-scale sheep farms. We collected 474 fecal samples and 312 environmental samples from Hu sheep on a large-scale sheep farm in Henan, China. The prevalence of *G. duodenalis* was determined by nested PCR targeting the β‑giardin (*bg*) gene. The assemblages and multilocus genotypes (MLGs) were investigated based on analyses of three genetic loci, i.e. *bg*, glutamate dehydrogenase (*gdh*), and triosephosphate isomerase (*tpi*). To detect mixed infections of different assemblages, assemblage A/E-specific PCRs were performed to amplify the *tpi* gene. The prevalence of *G. duodenalis* infection in sheep was 17.9% (81/474) and the positivity rate in environmental samples was 0.96% (3/312). Genetic analysis revealed the presence of two assemblages (assemblages A and E), with assemblage E being detected in both fecal and environmental samples, and assemblage A detected only in fecal samples. A total of 23 MLGs were obtained in fecal and environmental samples, all of which belonged to assemblage E. These results indicate the seasonal dynamics of *G. duodenalis* infection in sheep and environmental contamination on large-scale housing sheep farms and provide an important reference for the prevention and control of *G. duodenalis* on large-scale housing sheep farms.

## Abstract (fr)

*Giardia duodenalis* est un protozoaire parasitaire intestinal zoonotique commun et les moutons font partie de ses hôtes, mais peu d’informations sont disponibles sur les moutons élevés dans des stabulations à grande échelle. Le mouton Hu est une race de bétail locale protégée de première classe en Chine. Dans cette étude, nous avons étudié la dynamique saisonnière de l’infection à *G. duodenalis* chez les moutons Hu et la contamination environnementale des élevages ovins à grande échelle. Nous avons collecté 474 échantillons fécaux et 312 échantillons environnementaux de moutons Hu dans une ferme ovine à grande échelle dans le Henan, en Chine. La prévalence de *G. duodenalis* a été déterminée par PCR nichée ciblant le gène de la β‑giardine (*bg*). Les assemblages et les génotypes multilocus (MLG) ont été étudiés sur la base de l’analyse de trois locus génétiques, à savoir *bg*, glutamate déshydrogénase (*gdh*) et triosephosphate isomérase (*tpi*). Pour détecter des infections mixtes de différents assemblages, des PCR spécifiques aux assemblages A et E ont été réalisées pour amplifier le gène *tpi*. La prévalence de l’infection à *G. duodenalis* chez les ovins était de 17,9 % (81/474) et le taux de positivité dans les échantillons environnementaux était de 0,96 % (3/312). L’analyse génétique a révélé la présence de deux assemblages (assemblages A et E), l’assemblage E étant détecté à la fois dans les échantillons fécaux et environnementaux, et l’assemblage A détecté uniquement dans les échantillons fécaux. Au total, 23 MLG ont été détectés dans des échantillons fécaux et environnementaux, tous appartenant à l’assemblage E. Ces résultats montrent la dynamique saisonnière de l’infection à *G. duodenalis* chez les ovins et la contamination environnementale dans les élevages ovins à grande échelle et fournissent une référence importante pour la prévention et le contrôle de *G. duodenalis* dans les élevages ovins à grande échelle.

## Introduction

*Giardia duodenalis* is a typical intestinal parasitic protozoan that is most often spread through polluted food and water. Most animals and humans are susceptible to infection by this parasite, which hinders the reproductive ability of adult animals and the growth of young animals. Giardiasis, which is usually asymptomatic but can cause symptoms such as bloating, diarrhea, nausea, vomiting, and indigestion, affects more than 28.5 million people in China each year and is the second most common cause of infectious diarrhea in humans after viruses [5, 6, 13, 22]. The World Health Organization included giardiasis in the Neglected Diseases Initiative in 2006 [29].

Based on genetic analysis, *G. duodenalis* can currently be divided into eight assemblages (assemblages A–H) [15]. Most mammals can be infected by assemblages A and B, which are commonly categorized as zoonotic assemblages [16, 17, 26], while the other assemblages (C–H) are considered host-specific [11, 28]; however, this is a relative concept. Cats [24] and pigs [21] have been found with assemblage D, humans with assemblages C [31] and E [10], and pigs with assemblage F [1].

The dominant assemblage in sheep is assemblage E; assemblages A and B are also reported, of which the former is more common than the latter [18, 19, 25, 35]. Previous research has shown that the infection rates for *G. duodenalis* ranged from 0% to 64.11% [4, 22, 34] in sheep in China, and the potentially high infection rates in sheep mean that they are considered potential transmission sources for human infection.

The Hu sheep is a first-class protected local livestock breed in China with characteristics like heat and moisture resistance, early maturity, and good meat quality. The breed was listed in the *National Catalogue for Livestock and Poultry Genetic Resources* of the Ministry of Agriculture in 2000 and 2006. However, the occurrence of *G. duodenalis* on large-scale sheep farms, including those housing Hu sheep, has not been thoroughly investigated.

Multilocus genotyping (MLG) in *G. duodenalis* based on the beta-giardin (*bg*), triose-phosphate isomerase (*tpi*), and glutamate dehydrogenase (*gdh*) genes was first proposed to systematically analyze intra-assemblage genetic diversity [3]. Recent studies characterized *G. duodenalis* at individual loci, which often led to inconsistency in genotyping results [11].

Henan Province is located in the southern part of the North China Plain in the middle and lower reaches of the Yellow River, and most of the area is located in the warm temperate zone with four distinct seasons and simultaneous rain and heat. As an important grain growing base, and sheep farming is mostly intensive. The large breeding density leads to a favourable condition for the occurrence of *G. duodenalis* in sheep. In this work, the seasonal dynamics of *G. duodenalis,* based on environmental and fecal samples, were explored on a large-scale housed Hu sheep farm in Henan Province. In addition, based on MLG analysis, we aimed to identify the genetic features of *G. duodenalis* infection and environmental contamination of Hu sheep on large-scale sheep farms, to evaluate the danger of zoonotic transmission, and to specify the importance of this protozoan parasite for public health.

## Materials and methods

### Ethical standards

This study was conducted in accordance with the Chinese Laboratory Animal Administration Act (1988) after it was reviewed and its protocol approved by the Research Ethics Committee of Henan Agricultural University. Appropriate permission was gained from the animal owners before the collection of fecal sample specimens from the animals.

### Sample collection

A large-scale Hu sheep farm in Ruzhou City, Henan Province, China, with clear division of sheep physiological status, was selected. From March 2021 to January 2022, 786 samples, including 474 fecal samples and 312 environmental samples, were collected during four sessions, with one session per season (environmental samples were not collected in the spring for special reasons).

Sheep at different physiological stages were selected, including lactating lambs (1–20 days old), weaning lambs (1–3 months old), fattening lambs (4–6 months old), young lambs (7–12 months old), non-pregnant ewes, early pregnancy ewes (3 months gestation), late pregnancy ewes (1 week before parturition), lactating ewes, and breeding rams. Fresh feces from the animals were collected by rectal sampling, stored in clean containers, numbered, registered, and taken to the laboratory for storage at 4 °C until the DNA was extracted.

Sheep housing soil samples (5–30 g) were randomly collected at the entrance, fecal leakage board, exit, and corridor of the sheep house, put in clean containers, numbered, and register, delivered to the laboratory, and stored at 4 °C until the DNA was extracted.

### DNA extraction and PCR amplification

For DNA extraction, 100 mg of each fecal or environmental sample was put into a 1.5-mL centrifuge tube, and an E.Z.N.A Stool DNA kit (Omega Bio-Tek Inc., Norcross, GA, USA) was used to extract genomic DNA, according to the manufacturer’s instructions. The DNA was stored at −20 °C before use.

To investigate the presence of *G. duodenalis*, all DNA samples were tested by nested PCR targeting the *bg* gene. All *bg*-positive samples were further subjected to *gdh* and *tpi* gene amplification to analyze the MLGs of *G. duodenalis* and record mixed infections. To further understand the characteristics of the mixed infections, specific primers targeting the *tpi* locus were selected to identify assemblages A and E [14, 20, 27, 30, 34]. The PCR products were subjected to 1% agarose gel electrophoresis, developed with DNA Green (Tiandz, Beijing, China), and analyzed on a Tanon 3500 Gel Image Analysis System (Tanon, Shanghai, China).

### Sequencing and sequence analysis

All secondary positive PCR amplicons were purified and sequenced in both directions by a commercial company (SinoGenoMax Co. Ltd, Beijing, China). The obtained sequences were uploaded to seqman in DNAStar (http://www.dnastar.com/) to proofread the DNA chromatogram and compared and identified using Clustal X 2.0 (http://www.clustal.org/) and GenBank. Analysis of the amplification results for three genes (*bg*, *gdh*, *tpi*) allowed us to further conceptualize the genetic diversity of *G. duodenalis*.

### Statistical analysis

The chi square test in SPSS 22.0 (SPSS Inc., Chicago, IL, USA) was used to calculate differences in the incidence of *G. duodenalis* among sheep of different physiological states, samples from different enclosure environments, and samples from different seasons. Differences were considered statistically significant when *p* < 0.05.

### Phylogenetic analysis

To study the relationship between different isolates in more detail, phylogenetic analyses were performed using a concatenated dataset of *bg*, *tpi,* and *gdh* sequences with the MLGs of *G. duodenalis*.

The tandem sequence (*bg*–*tpi*–*gdh*) was used for phylogenetic reconstruction using Neighbor-Joining analysis, and the genetic distances were calculated in the Kimura 2-parameter model in MEGA10 (https://www.megasoftware.net/). Bootstrap analysis (1000 replicates) was used to evaluate the reliability of the phylogenetic tree.

### Seasonal meteorological information

Information on the average temperature and precipitation data over the four seasons in Ruzhou was obtained through the *Chinese Central Meteorological Station* (http://www.nmc.cn/).

### Nucleotide sequence accession numbers

The NCBI’s GenBank database has received a deposit of representative nucleotide sequences from this study, and the accession numbers are OP142425–OP142427 for the *bg* gene, OP142428–OP142431 for the *gdh* gene, and OP156823–OP156824 for the *tpi* gene.

## Results

### *Giardia duodenalis* infection in Hu sheep and contamination of the environment

Overall, 81 (17.09%, 95% CI: 13.69–20.49%) fecal samples and 3 (0.96%, 95% CI: 0.13–2.05%) environmental samples were *G. duodenalis*-positive (Table 1).

**Table 1:** Prevalence of *G. duodenalis* in Hu sheep and rates of positive environmental samples from different seasons.

Sample type	Sampling time	Tested	No. positive (%)	*p*-value	Genotype or subtype (*n*)		
					*bg*	*gdh*	*tpi*
Fecal sample	Spring	135	41 (30.37)	Ref. group	A (3), E (38)	E (32)	A (3), E (28), A + E (8)
	Summer	106	10 (9.43)	<0.01	E (10)	E (9)	E (9), A + E (2)
	Autumn	129	10 (7.75)	<0.01	E (10)	E (9)	E (8), A + E (1)
	Winter	104	20 (19.23)	<0.01	A (3), E (17)	E (19)	E (10), A + E (1)
Total		474	81 (17.09)		A (6), E (75)	E (69)	A (3), E (55), A + E (12)
Environmental sample	Summer	108	3 (2.78)	Ref. group	E (3)	E (3)	E (3)
	Autumn	108	0 (0.00)	>0.05	–	–	–
	Winter	96	0 (0.00)	>0.05	–	–	–
Total		312	3 (0.96)		E (3)	E (3)	E (3)

Among the fecal samples, the rate of parasite-positive samples was the highest in spring (30.37%) and the lowest in autumn (7.75%). The rates of positive samples in winter and summer were 19.23% (20/104) and 9.43% (10/106), respectively. The rate of positive samples differed among the seasons, and all differences between seasons were statistically significant (*p* < 0.05), except for between summer and autumn (*p* > 0.05). The differences between spring, summer, and autumn, and the difference between winter and autumn were highly significant (*p* < 0.01). In environmental samples, *G. duodenalis* was only detected in summer (all positive samples were taken from the fecal leakage board), and there were no significant differences between these environmental samples and those in other seasons (*p* > 0.05) (Table 1).

On analyzing the presence of the parasites in Hu sheep of different physiological stages, we discovered that the highest infection rates of *G. duodenalis* occurred in weaning lambs (43.08%), and lactating ewes had the lowest infection rates (4.23%). The prevalence of *G. duodenalis* in late-pregnancy ewes (8.62%) was lower than that in early-pregnancy ewes (20.37%) and non-pregnant ewes (22.73%), while the prevalence of other groups ranged from 5.00% to 22.73%. The *G. duodenalis* infection rate of weaned lambs was significantly higher than that of Hu sheep of other physiological stages (*p* < 0.05) (Table 2).

**Table 2:** Prevalence of *G. duodenalis* in Hu sheep of different physiological stages.

Physiological state	Sample size	No. positive (%)	*p*-value	Assemblage (*n*)
Lactating lambs	39	5 (12.82)	<0.01	E (5)
Weaning lambs	65	28 (43.08)	Ref. group	E (24), A + E (4)
Fattening lambs	51	9 (17.65)	<0.01	E (8), A + E (1)
Young lambs	40	2 (5.00)	<0.01	A + E (2)
Non-pregnant ewes	66	15 (22.73)	<0.05	E (10), A + E (5)
Early pregnancy ewes	54	11 (20.37)	<0.01	E (7), A + E (4)
Late pregnancy ewes	58	5 (8.62)	<0.01	E (5)
Lactating ewes	71	3 (4.23)	<0.01	E (3)
Breeding rams	30	3 (10.00)	<0.01	E (1), A + E (2)
Total	474	81 (17.09)		E (63), A + E (18)

### *Giardia duodenalis* assemblage distributions

In the nested PCR analysis of the *bg* gene, out of 81 *G. duodenalis-*positive fecal samples, six were zoonotic assemblage A and 75 were assemblage E, and all three positive environmental samples belonged to assemblage E.

When the 84 *bg*-positive samples were analyzed based on the *gdh* gene, 69 of the fecal samples and all 3 environmental samples belonged to assemblage E. For *bg*-positive samples, DNA sequence analysis of the *tpi* locus revealed the occurrence of assemblage A (15) and assemblage E (67) in fecal samples and assemblage E (3) in environmental samples, and assemblages A and E co-occurred in 12 fecal samples (Table 1).

### Subtypes of assemblages A and E

A total of 84, 72, and 85 sequences were obtained for the *bg*, *gdh*, and *tpi* loci, respectively, from Hu sheep and barn environment samples (Table 1).

Using KT922248 as the reference sequence for the *bg* locus, the 78 assemblage E *bg* locus sequences were identified as belonging to eight subtypes, including five known subtypes (E1, E2, E3, E5, and E11) and three new subtypes (E47, E48, and E49) (Table 3). The 72 assemblage E *gdh* locus sequences were classified into eight subtypes according to GenBank sequence KY711410, including four known (E46, E50, E51, and E52) and four new subtypes (E53, E54, E55, and E56) (Table 3). Four subtypes were identified from the 70 assemblage E *tpi* locus sequences using the reference sequence MF095054, including three previously unnamed subtypes, E57, E58, and E59, and one novel subtype, E60 (Table 3) [23].

**Table 3:** Intra-assemblage substitutions of assemblage E at the *bg*, *gdh*, and *tpi* loci.

Subtypes	Nucleotide at position							GenBank ID	No. positive	
									Fecal sample	Environmental sample
*bg*										
	53	65	119	170	381	413				
Ref. sequence	C	C	C	A	G	T		KT922248		
E1	–	–	–	–	–	C		MK610388	38	2
E2	–	T	–	–	–	–		KP635113	1	0
E3	–	T	–	–	–	C		KP635114	3	0
E5	–	–	–	–	–	–		KY633466	27	1
E11	–	T	–	G	–	C		KY633471	1	0
E47	–	–	T	–	–	–		OP142427	3	0
E48	T	–	–	–	–	–		OP142426	1	0
E49	–	–	–	–	T	C		OP142425	1	0
*gdh*										
	21	129	168	243	318	333	384			
Ref. sequence	C	C	G	C	C	A	C	KY711410		
E50	–	–	–	–	–	–	–	KY711410	25	1
E46	–	–	A	T	–	–	–	MT123526	1	0
E51	–	–	A	–	–	–	–	AB692774	33	1
E52	–	–	–	–	A	–	–	KP635107	1	0
E53	–	G	A	–	–	–	–	OP142428	5	0
E54	T	–	–	–	–	–	–	OP142429	2	0
E55	–	–	–	T	–	–	–	OP142430	2	0
E56	–	–	A	–	–	G	T	OP142431	0	1
*tpi*										
	91	168	262							
Ref. sequence	G	C	T					MF095054		
E57	–	–	–					MF095054	1	0
E58	A	–	–					MH230888	28	1
E59	A	–	C					MK442915	33	2
E60	A	T	C					OP156823	5	0

Using GenBank sequence KJ027408 as a reference, six *bg* locus sequences were identified, three of subtype AI and three with two single-nucleotide polymorphisms, named A1. Similarly, the GenBank sequence FJ560569 was utilized as a reference, and the 15 sequences at the *tpi* locus were used to classify the parasites into three subtypes, including two known subtypes (A4 and A6) and a novel subtype A8 (Table 4).

**Table 4:** Intra-assemblage substitutions of assemblage A at the *bg* and *tpi* loci.

Subtypes	Nucleotide at position		GenBank ID	No. positive	
				Fecal sample	Environmental sample
*bg*					
	68	317			
Ref. sequence	C	C	KJ027408		
AI (16)	–	–	KR075937	3	0
A1 (22)	T	T	MK610391	3	0
*tpi*					
	223	353			
Ref. sequence	A	A	FJ560569		
A4 (9)	–	–	MK639172	8	0
A6 (7)	G	–	MN704937	6	0
A8 (56)	–	G	OP156823	1	0

### Multilocus genotyping

**Figure 1:**
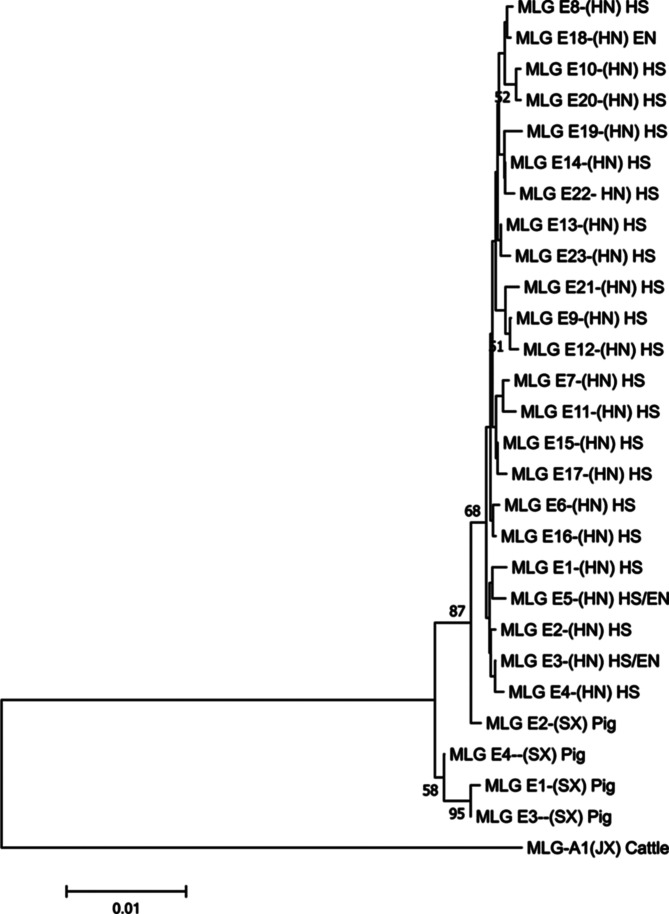
Phylogenetic relationships among *G. duodenalis* MLGs. The phylogenetic tree was constructed using a concatenated dataset of the *bg*, *tpi,* and *gdh* gene sequences and Neighbor-Joining analysis with the Tamura–Nei model. MLGs with unambiguous sequences identified in this study (Table 5) were analyzed with reference isolates. Bootstrap values greater than 50% from 1000 replicates are shown at the nodes. HN: Henan; SX: Shaanxi; JX: Jiangxi; HS: Hu sheep; EN: Environment.

Altogether, 62 specimens were simultaneously amplified at the three genetic loci, comprising 59 fecal samples and 3 environmental samples. The sequences obtained at all three loci in 54 of the 59 fecal samples belonged to assemblage E, forming 22 new assemblage E MLGs (MLGs E1 to E17 and MLGs E19 to E23). The sequences obtained from the 3 environmental samples formed 3 MLGs (MLGs E3, E5, and E18). Other samples were mixed infections of assemblages A and E (Table 5). Phylogenetic analysis showed that all assemblage E MLGs were significantly different from those reported previously [23, 33] for *G. duodenalis* (Fig. 1).

**Table 5:** Multilocus sequence genotyping of *G. duodenalis* in Hu sheep in Henan Province using the *bg*, *tpi,* and *gdh* genes.

Isolate/specimen ID	Genotype			MLGs (*bg*–*tpi*–*gdh*)
	*bg*	*gdh*	*tpi*	
71	E1	E52	E59	MLG-E1
110, 120, 125, 133, 786	E1	E46	E58	MLG-E2
35, 40, 59, 61, 261, 340a, 522	E1	E46	E59	MLG-E3
526	E1	E46	E60	MLG-E4
65, 365a, 496	E1	E51	E58	MLG-E5
11, 19, 68, 252, 256, 519, 769, 822	E1	E51	E59	MLG-E6
127	E1	E55	E58	MLG-E7
114	E1	E54	E58	MLG-E8
112	E1	E54	E59	MLG-E9
108, 116	E1	E54	E60	MLG-E10
56	E2	E53	E58	MLG-E11
282	E3	E50	E58	MLG-E12
60, 109, 115, 119, 540, 788, 793	E5	E46	E58	MLG-E13
52, 262, 486	E5	E46	E59	MLG-E14
113, 257	E5	E51	E58	MLG-E15
254, 263, 463, 536, 823, 826	E5	E51	E59	MLG-E16
825	E5	E51	E57	MLG-E17
497	E5	E54	E58	MLG-E18
317^a^	E5	E56	E59	MLG-E19
106	E5	E53	E60	MLG-E20
128	E11	E46	E58	MLG-E21
266	E47	E46	E59	MLG-E22
118	E48	E46	E58	MLG-E23
7	E1	E50	A6	Mixed 1 (A + E)
19, 34	E1	E51	A6	Mixed 2 (A + E)
59	E1	E46	A4	Mixed 3 (A + E)
108	E1	E54	A6	Mixed 4 (A + E)
127	E1	E55	A4	Mixed 5 (A + E)
56	E2	E53	A8	Mixed 6 (A + E)
106	E5	E53	A6	Mixed 7 (A + E)
262	E5	E46	A4	Mixed 8 (A + E)
463	E5	E51	A4	Mixed 9 (A + E)
266	E47	E46	A4	Mixed 10 (A + E)
9	E49	E51	A4	Mixed 11 (A + E)
57	E5	–	A8	Mixed 12 (A + E)
106	E1	–	A4	Mixed 13 (A + E)
813	AI	E51	E59	Mixed 14 (A + E)
817	AI	E46	E59	Mixed 15 (A + E)
16	AI	–	E59	Mixed 16 (A + E)
22	A1	E50	–	Mixed 17 (A + E)
51	A1	–	E59	Mixed 18 (A + E)

a Environmental sample.

N-dash (–) indicates that no data were obtained.

### Weather information for all seasons in Ruzhou

Ruzhou City, Henan Province, has a warm temperate semi-humid continental monsoon climate with four distinct seasons: spring (temperature: 9.47–21.27 °C, precipitation: 42.47 mm), summer (temperature: 21.60–31.20 °C, precipitation: 109.97 mm), autumn (temperature: 10.63–20.97 °C, precipitation: 46.37 mm) and winter (temperature: −1.6 to 8.2 °C, precipitation: 11.77 mm).

## Discussion

This paper reports, for the first time, the seasonal dynamics of *G. duodenalis* infections in Hu sheep and environmental contamination of large-scale housing sheep farms in China. The results showed that the incidence of *G. duodenalis* in Hu sheep was 17.09% (81/474), and in environmental samples, it was 0.96% (3/312). The prevalence of *G. duodenalis* in Hu sheep was higher than the previously reported prevalence of *G. duodenalis* infections in sheep in Henan Province (1.82–5.64%) [22]; however, it was far lower than that in Inner Mongolia (64.11%) [4]. This variation may be due to a range of factors, including the presence of other animal species on the farm, farming practices and size, inspection methods, study design, number of samples analyzed, timing of sample collection, and environmental conditions.

*Giardia* cysts can survive for months in high-humidity, low-temperature, low-sunlight-level, and low-salinity environments [9, 11]. Ruzhou City, Henan Province, has a warm temperate semi-humid continental monsoon climate with four distinct seasons: it is warm in spring, hot in summer, cool in autumn, and cold in winter. This may explain why *G. duodenalis* infection rates are highest in spring (30.37%). Furthermore, the relatively cold winter temperatures, as well as the dry air in Ruzhou, may be the reason why the infection rate is lower in winter (19.23%) than spring. It is possible that the summer is not suitable for *G. duodenalis* transmission, resulting in lower infection rates (9.43%), and the influence of the summer results in lower infection rates in the autumn (7.75%).

A literature search shows that most reports describe a negative correlation between *G. duodenalis* infection rates and age [7, 32, 36], which is supported by our findings. Not all lambs had higher infection rates than adult sheep, but the rates of *G. duodenalis* were the highest in weaning lambs (43.08%, 28/65), which may be because weaning is a period of transition of feeding methods, and the lambs’ immunity is low because of stress. Moreover, the parasite prevalence in lactating lambs (12.82%, 5/39) was lower than that in fattening lambs (17.65%, 9/51), probably because the lactating lambs were fed by fewer infected lactating ewes, reducing the probability of cross-infection. It was also found that the infection rate of late-gestation ewes was lower than that of early-gestation ewes and non-pregnant ewes. This phenomenon may be attributable to pregnant ewes being fed a more varied diet than their non-pregnant counterparts. Furthermore, pregnant ewes are typically better cared for and housed in more comfortable, quiet, and secure enclosures. Because of interactions among these factors, pregnant ewes may have had higher immunity and disease resistance, resulting in lower infection rates than non-pregnant ewes.

In this study, 312 environmental samples were collected. Although only three positive samples were found in the summer (all on the fecal leakage board), the results suggest that *G. duodenalis* was present in the environment. Because of the high density of livestock on large-scale sheep farms, *G. duodenalis* in the environment is likely to be consumed by sheep, allowing the transmission cycle to continue [2]. The wastewater used for cleaning fences is also at risk of entering the irrigation water used for vegetables, thus increasing the infection risk for humans and other animals [8, 12].

Available studies suggest that assemblage E is the dominant assemblage of *G. duodenalis* in sheep, while assemblages A and B have also been occasionally reported [22]. The results of this survey are the same as those of other surveys. Based on nested PCR amplification of *bg*, *gdh,* and *tpi*, assemblage E was still the dominant cluster of *G. duodenalis* in Hu sheep, followed by assemblage A, while assemblage B was not found. A total of 18 samples were detected with mixed infections of assemblages A and E using specific primers for the *tpi* locus of these assemblages. Because commonly used primers for the *tpi* locus were unable to detect two or more assemblages in the same sample in the past, it can be inferred that the identification of assemblage A and mixed infections in previous investigations were relatively low because of the specificity of the primers [30]. The public health impact of assemblage A, a common human–animal *G. duodenalis* assemblage, and the risk of sheep acting as potential *G. duodenalis* conservator hosts have been simultaneously underestimated.

To further reveal the genetic variations in assemblages A and E, another two genetic loci (*gdh* and *tpi*) of 84 *bg*-positive samples were analyzed with high resolution via the multilocus genotyping tool. In the fecal samples from Hu sheep, seven assemblage E subtypes were identified using the *gdh* locus, and four assemblage E subtypes as well as three assemblages A subtypes were identified using the *tpi* locus. In the environmental samples, three assemblage E subtypes were identified using the *gdh* locus, and two assemblage E subtypes were identified using the *tpi* locus (Tables 3 and 4). In addition, 14 samples contained different assemblages at the three loci (Table 5). Twenty-three MLGs at all three loci of assemblage E were successfully amplified from 57 samples with the same assemblage genotype. These findings suggest that there is high subtype diversity and genetic variation within assemblage E.

## Conclusions

This dynamic survey of *G. duodenalis* in fecal and environmental samples collected in different seasons from a large-scale sheep farm in Henan Province revealed the prevalence of *G. duodenalis* in Hu sheep and environmental contamination. The prevalence of *G. duodenalis* in Hu sheep was 17.09% (81/474) and the rate of *G. duodenalis* in the environment was 0.96% (3/312), indicating that the prevalence of the protozoa in Hu sheep is high and it can be transmitted through the environment. Large-scale farms with a high density of sheep should take measures to avoid horizontal transmission of *G. duodenalis*. In addition, mixed infections of assemblages A and E in Hu sheep were more severe than reported by previous studies, suggesting that the presence of assemblage A has been underestimated. Therefore, the significance of *G. duodenalis* in sheep for public health may need to be re-examined.
